# Supplementary material for: Analysis of the Circulating Metabolome of Patients with Cutaneous, Mucosal and Uveal Melanoma Reveals Distinct Metabolic Profiles with Implications for Response to Immunotherapy
Source: Cancers (Basel). 2023 Jul 21;15(14):3708. doi: 10.3390/cancers15143708 (PMC10378038; doi:10.3390/cancers15143708)
Supplement: Supplementary file 1 [file cancers-15-03708-s001.zip › cancers-2444130-supplementary.pdf]

Supplementary Material

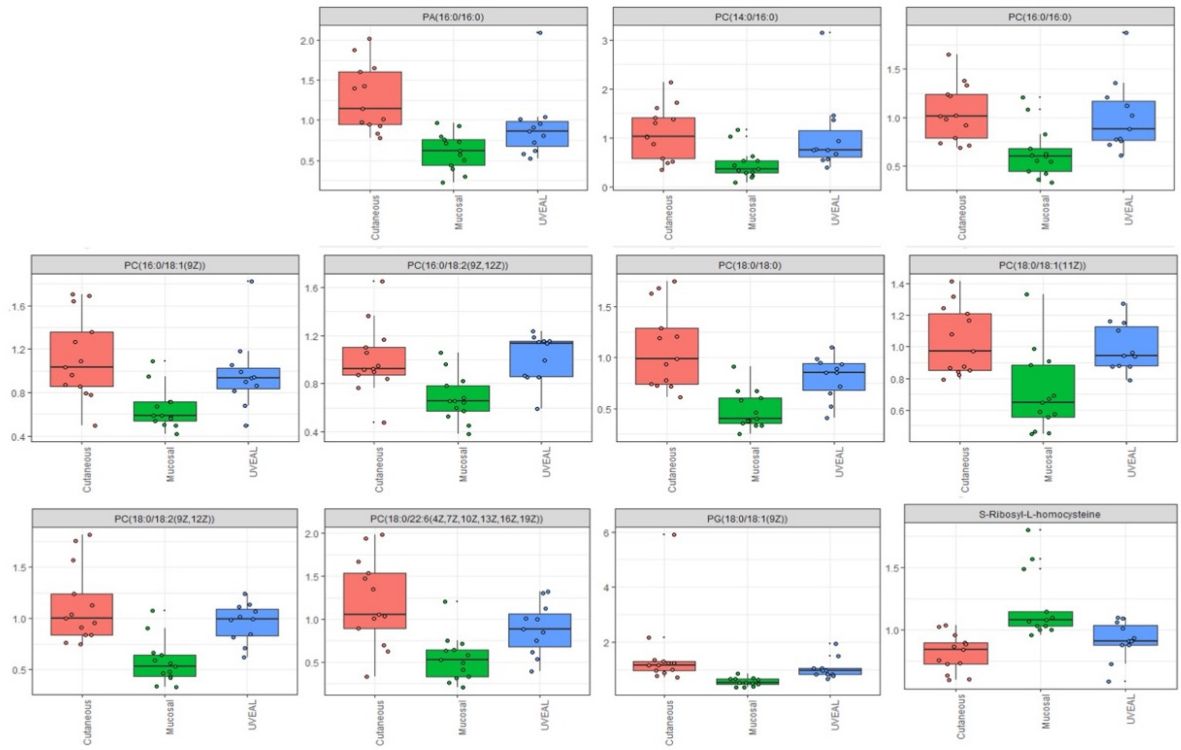

Figure S1: Characterization of phosphatidic acid (PA), phosphatidylcholine (PC), glycerophospholipid (PG), and s-ribosyl-l-homocysteine according to the melanoma subtypes.

Median Overall Survival (m)	
**SPM < 3.54**	**SPM ≥ 3.55**
**12.8**	**6.1**
HR: 0.478 95% CI 0.13 to 1.72 p = 0.248	

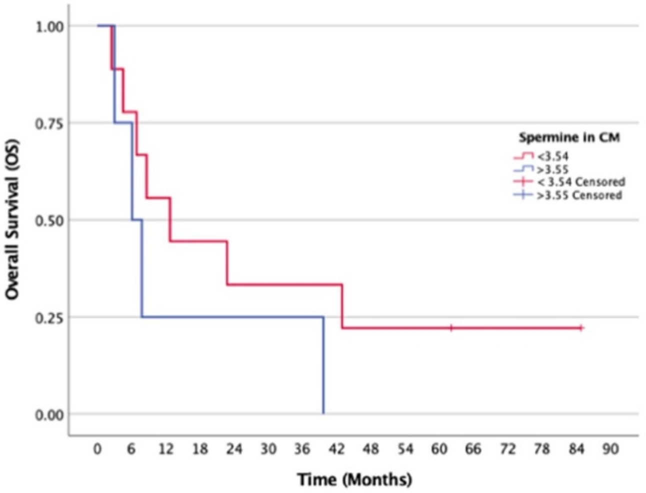

Figure S2: Overall survival of cutaneous melanoma patients according to SPM levels. SPM cut-off point used for analysis was in the 70th percentile.
